# Supplementary material for: Effects of Vitamin A on Yanbian Yellow Cattle and Their Preadipocytes by Activating AKT/mTOR Signaling Pathway and Intestinal Microflora
Source: Animals (Basel). 2022 Jun 7;12(12):1477. doi: 10.3390/ani12121477 (PMC9219514; doi:10.3390/ani12121477)
Supplement: Supplementary file 1 [file animals-12-01477-s001.zip › animals-1728887-supplementary.pdf]

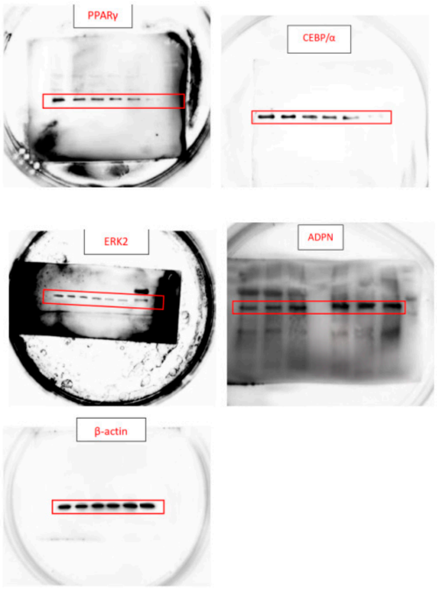

Figure S1. Original Western blot figures of PPARγ, C/EBPα, ERK2, and ADPN in bovine adipocytes treated with ATRA.

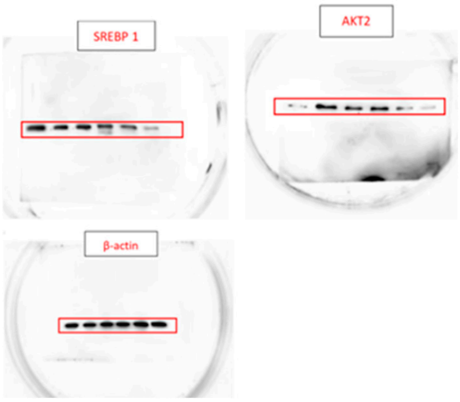

Figure S2. Original Western blot figures of SREBP1 and AKT2 in bovine adipocytes treated with ATRA.

Table S1. Image J analysis of grayscale values.

GENE mol/L	0	2×10^{-7}	1×10^{-6}	2×10^{-6}	1×10^{-5}	2×10^{-5}
ADPN	73,455	75,997	82,773	82,810	82,846	83,065
AKT2	36,202	30,359	25,578	13,926	6280	4433
CEBP/α	38,659	25,664	25,455	20,296	19,516	9123
ERK2	49,245	32,330	24,829	21,681	12,689	13,751
PPARγ	77,768	64,919	48,210	43,268	34,658	4188
SREBP 1	45,050	42,438	41,284	39,835	33,890	13,593
β-actin	29,899	28,914	31,077	32,454	35,458	35,857
